# Types of menopausal experiences and perceptions of menopause-related health care among multicultural women: a Q methodology study

**DOI:** 10.4069/whn.2026.08.31

**Published:** 2026-09-29

**Authors:** Sun Jung Park, Eun Young Choi

**Affiliations:** 1 Department of Nursing, Sahmyook University, Seoul, Korea; 2 Department of Nursing, Kyungdong University, Wonju, Korea

**Keywords:** Health behavior, Health knowledge, Menopause, Multicultural women

## Abstract

**Purpose:**

This study used Q methodology to identify and classify multicultural women’s subjective perceptions of menopausal experiences and menopause-related health care, with the aim of providing foundational data for culturally tailored nursing interventions.

**Methods:**

Q methodology was used to identify patterns in subjective perceptions of menopausal experiences and health management. A literature review and in-depth interviews yielded 34 Q-statements. Forty multicultural women aged 40–60 years living in Korea completed Q-sorting from March 15 to April 5, 2026. The data were analyzed by principal component factor analysis using PQMethod.

**Results:**

Four types were identified: (1) symptom distress recognition, characterized by a strong awareness of physical discomfort; (2) health management interest, characterized by active efforts to obtain health information and professional consultation; (3) menopausal acceptance and self-management, characterized by an emphasis on proactive self-care; and (4) emotional change and social support awareness, characterized by emotional fluctuations and an emphasis on family support. Together, the four types explained 44.99% of the total variance. Reports of musculoskeletal pain and emotional symptoms, including loneliness and depression, were common across all four types.

**Conclusion:**

Cultural background, migration experience, health literacy, and social support shape the multidimensional menopausal experiences and health care perceptions of multicultural women. Meeting this population’s diverse needs requires tailored, culturally sensitive nursing interventions, better access to health care, and family-centered support programs.

## Introduction

For women, middle age marks a transition involving biological aging and the cessation of reproductive function [1]. Menopausal symptoms during this period can substantially affect physical, psychological, and social well-being [2,3]. Common physical symptoms include hot flashes, night sweats, sleep disturbances, and musculoskeletal pain; psychological symptoms may include depression, anxiety, emotional instability, and cognitive changes [2,3]. How women experience and interpret menopause, however, varies with cultural beliefs, social support, health literacy, and individual health perceptions [1-3].

As Korea’s multicultural population has grown, the number of multicultural women has steadily increased; most initially entered the country through marriage to Korean nationals. International marriages between Korean men and foreign-born women began to rise sharply in the mid-1990s and continued to increase through the 2000s, when they accounted for close to 10% of all marriages registered nationwide each year [4]. The aging of women who married during this early period of migration has shifted the age distribution of multicultural women in Korea. According to the 2021 National Survey on Multicultural Families, spouses in their 40s and 50s now account for more than two-thirds of all married immigrants and naturalized citizens, indicating that a substantial and growing proportion is entering the menopausal transition [5]. Previous studies have found that language barriers, cultural differences, social isolation, limited social support, and difficulty accessing health care services make multicultural women more vulnerable to health problems than native Korean women [6-8]. In midlife, the demands of adapting to a new cultural environment may coincide with the menopausal transition, creating more complex physical and psychosocial health needs [7]. Nevertheless, most menopause-related studies have focused on the general female population or Korean women [9,10], leaving the menopausal experiences of multicultural women relatively underexplored.

Perceptions of menopause and related health behaviors encompass more than biological responses to hormonal changes. Cultural attitudes toward aging and femininity, health beliefs, previous health care experiences, and social context also shape these perceptions and behaviors [3,11]. Multicultural women often experience menopause in a bicultural environment where values from their countries of origin coexist with Korean health care norms and expectations. They may therefore view menopause as a natural life stage, a medical condition requiring treatment, or a private matter that should not be discussed openly [3,11]. These interpretations can affect how women manage symptoms, use health care, and engage in health-promoting behaviors.

Despite growing interest in the health of multicultural populations, previous studies have predominantly used quantitative approaches to describe menopausal experiences through average scores or statistical associations [9,10]. Such studies provide valuable information but may not fully capture the range of subjective viewpoints and meanings that multicultural women attach to menopause and health management. The close relationship of menopausal experiences to personal beliefs, cultural values, and individual life experiences calls for an approach that systematically explores subjectivity [12-15].

In this study, menopausal experiences encompass the multidimensional physical, psychological, emotional, and social changes that women perceive during the menopausal transition and postmenopausal period. Menopause-related health care perceptions encompass subjective beliefs, attitudes, and perceptions about symptom management, seeking health information, using health care, participating in health education, and practicing self-management in relation to menopause.

Because these perceptions influence how women interpret menopausal changes and respond to health needs [3,11], understanding their subjective structure is essential to developing culturally appropriate interventions for multicultural women. This study therefore used Q methodology to identify and classify multicultural women’s subjective perceptions of menopausal experiences and menopause-related health care.

Q methodology investigates human subjectivity by identifying viewpoints shared among individuals [14,15]. Combining qualitative and quantitative methods, it systematically classifies subjective perceptions into meaningful types even in relatively small samples [13-15]. Its focus on understanding diverse perspectives rather than estimating the prevalence of opinions [16] makes it particularly useful for exploring health perceptions and experiences in culturally diverse populations. Previous studies have successfully used Q methodology to examine subjective health beliefs, illness experiences, and attitudes toward health care in various populations [10-12].

This study therefore used Q methodology to identify and classify subjective perceptions of menopausal experiences and menopause-related health care among multicultural women living in Korea. By examining the perceptual structures underlying menopause and health management, the study sought to improve understanding of middle-aged multicultural women’s health needs. The findings may inform culturally sensitive nursing interventions, health education programs, and community-based support strategies tailored to this population’s unique characteristics. They are also expected to contribute to evidence-based women’s health nursing practice and health policy development intended to improve multicultural women’s health and quality of life.

## Methods

**Ethics statement:** The Institutional Review Board of Sahmyook Health University approved this study (IRB-2-70094958-A-N-01-260223-HR-001-01). All participants provided informed consent.

### Purpose of the study

This study used Q methodology to identify and classify multicultural women’s subjective perceptions of menopausal experiences and health care and to examine the characteristics of each type. Exploring their views on menopause and health management was intended to provide data for culturally sensitive nursing interventions and health promotion programs tailored to their specific needs. The findings are also expected to improve understanding of multicultural women’s health experiences and support evidence-based nursing practice and policy development for this population.

### Research design

This exploratory study used Q methodology to investigate multicultural women’s subjective perceptions of menopausal experiences and health care and classify the underlying perceptual structures. Q methodology systematically examines human subjectivity through qualitative interpretation and quantitative factor analysis. It allows researchers to identify shared viewpoints and understand participants’ diverse value systems and perceptions [14,15].

The study followed the Q-methodology reporting checklist proposed by Churruca et al. [17] to report Q-sample development, data collection, and analytical procedures transparently.

Multicultural women experience menopause in distinct cultural and social contexts, so their perceptions of menopause and health care may vary substantially with personal beliefs, migration experiences, and cultural background. Q methodology was therefore used to identify and interpret types of subjective perceptions of menopausal experiences and menopause-related health care in this population and to explore each type’s characteristics.

### Research methodology

The study comprised five stages, beginning with construction of the Q-population and Q-sample and ending with interpretation of the types (Figure 1).

#### Construction of the Q-population and Q-sample

The Q-population was developed to capture the full range of multicultural women’s subjective perceptions of menopausal experiences and health care. To represent cultural diversity, the literature review covered menopausal experiences, health beliefs, health care utilization, and cultural perceptions of menopause among women from different cultural backgrounds. In-depth interviews with multicultural women from different countries of origin also elicited culturally diverse experiences and perspectives on menopause and health care. Statement development placed particular emphasis on culturally influenced beliefs about menopause, family roles, help-seeking behaviors, health care utilization, and social support.

Statements were initially collected through an extensive review of domestic and international literature on menopause, multicultural women’s health, health-seeking behaviors, and health care utilization. Semi-structured, in-depth interviews with five multicultural women aged 40–60 years supplemented the review by capturing experiences that might be underrepresented in the literature.

The literature review and in-depth interviews initially yielded 128 statements: 93 from the review and 35 from the interviews. Removing duplicates, integrating similar content, and clarifying wording reduced this set to 62 statements. The remaining statements were grouped into five domains: (1) perceptions of menopausal symptoms, (2) emotional experiences, (3) health-seeking behaviors, (4) health care utilization experiences, and (5) cultural beliefs and social support.

To establish content validity, an expert panel of two nursing professors and one women’s health nursing specialist reviewed the preliminary statements. The panel assessed each statement for relevance, clarity, redundancy, and representativeness. Its recommendations guided the revision, merging, or removal of statements. The 34 statements that best represented the range of perceptions across the five domains were selected for the final Q-sample.

#### Selection of the participant sample

The participant sample comprised 40 multicultural women aged 40–60 years living in Korea. Q methodology seeks to identify patterns of subjectivity rather than achieve statistical representativeness. Sample size is therefore generally determined by the number of Q-statements rather than the population size. Previous Q-methodology studies have recommended 20–60 participants for Q-samples of approximately 30–40 statements [14-16].

With 34 statements in the final Q-sample, 40 participants were considered sufficient to identify stable, meaningful perception types while preserving diversity of viewpoints.

Purposive sampling was used to recruit participants with varying nationalities, lengths of residence in Korea, educational levels, and health care experiences. Recruitment included women from Vietnam, the Philippines, Cambodia, and China to ensure cultural diversity. This approach was intended to capture a broad range of culturally influenced perceptions and experiences of menopause and health care. Participant selection therefore considered differences in cultural beliefs about menopause, family roles, help-seeking behaviors, and health care utilization as important sources of variation in subjective perceptions.

The inclusion criteria were as follows:

• Multicultural women currently living in Korea;

• Aged 40–60 years, with experience of menopausal symptoms or perceived menopause-related physical or emotional changes;

• Sufficient Korean proficiency to understand study instructions, read and comprehend Q-statements, complete Q-sorting independently, and explain their statement selections verbally during the post-sorting interview;

• Ability to provide informed consent voluntarily.

To establish adequate communication ability, participants had to demonstrate that they could understand the study explanation, read Q-statements, and verbally explain their selections during the post-sorting interview. Severe cognitive impairment or significant communication difficulties were grounds for exclusion.

#### Q-sorting procedure

Participants completed Q-sorting individually in a quiet setting with 34 statement cards. Before sorting, they received a detailed explanation of the study’s purpose and the Q-sorting procedure.

Participants first read all 34 statements and provisionally classified them as agree, neutral, or disagree. They then arranged the statements in a forced quasi-normal distribution from −4 (most strongly disagree) to +4 (most strongly agree), according to their degree of agreement with each statement.

The 34 statements were distributed across the framework as follows: two at +4, three at +3, four at +2, five at +1, six at 0, five at −1, four at −2, three at −3, and two at −4.

The forced distribution encouraged participants to prioritize statements according to their own viewpoints and helped identify shared patterns of perception.

After completing the Q-sort, participants explained in interviews why they had placed particular statements at the two extremes (+4 and −4). Data were collected from March 15 to April 5, 2026.

#### Data analysis and interpretation

Q-sort data were analyzed with PQMethod ver. 2.35 (Peter Schmolck, Neubiberg, Germany). Principal component factor analysis was used to identify patterns of subjectivity. Factors with eigenvalues greater than 1.0 were retained and subjected to varimax rotation to aid interpretation. Each factor was interpreted using standardized scores (Z-scores), participant characteristics, and qualitative interview data. Quantitative and qualitative findings were then interpreted together to characterize each perception type and establish its meaning.

## Results

### General characteristics of the participants

Of the 40 participants, the largest proportion were Vietnamese (n=18, 45.0%), followed by Filipino (n=15, 37.5%), Cambodian (n=5, 12.5%), and Chinese (n=2, 5.0%). The largest age group was 40–44 years (n=18, 45.0%), followed by 50–54 years (n=11, 27.5%), 45–49 years (n=9, 22.5%), and 55 years or older (n=2, 5.0%).

Eighteen participants (45.0%) had lived in Korea for 10–15 years, 15 (37.5%) for 5–10 years, five (12.5%) for more than 15 years, and two (5.0%) for less than 5 years. Marriage duration was 5–10 years for 21 participants (52.5%), more than 15 years for 10 (25.0%), and 10–15 years for nine (22.5%).

Most participants were unemployed (n=31, 77.5%). Twenty-six (65.0%) reported no specific menopausal symptoms but perceived general physical or emotional changes associated with menopause, seven (17.5%) were unsure, and seven (17.5%) reported specific symptoms. Fifteen participants (37.5%) reported receiving menopausal health information, whereas 25 (62.5%) did not. Thirty-one (77.5%) reported using hospitals or public health centers for health concerns. Eighteen participants (45.0%) perceived no family support, 13 (32.5%) perceived very high support, and nine (22.5%) perceived moderate support (Table 1).

### Identification of Q-types

Q-factor analysis identified four distinct types of subjective perceptions of menopausal experiences and menopause-related health care among multicultural women. Together, the four factors explained 44.99% of the total variance. The proportions explained were 18.85% for Type I (symptom distress recognition), 11.00% for Type II (health management interest), 8.63% for Type III (menopausal acceptance and self-management), and 6.52% for Type IV (emotional change and social support awareness) (Table 2).

Correlations among the four types were relatively low, with coefficients ranging from −.10 to .23, suggesting that each type represented an independent pattern of subjective perception (Table 3).

### Characteristics of the types

The four types comprised 13 participants in Type I, 11 in Type II, nine in Type III, and seven in Type IV. Each type was interpreted using statements indicating strong agreement (Z-score ≥+1.00) or strong disagreement (Z-score ≤−1.00), along with participants’ demographic characteristics and qualitative comments (Table 4).

#### Type I: Symptom distress recognition

Women in Type I expressed strong agreement with “It is difficult to sleep well after menopause” (Z=2.13), “Menopausal symptoms sometimes make daily life difficult” (Z=1.94), “My joints or muscles ache after menopause” (Z=1.60), and “I get tired easily after menopause” (Z=1.36). Their agreement that menopause is a natural process experienced by women was also strong (Z=1.33).

By contrast, women in this group disagreed with statements emphasizing health education, social support, and positive reinterpretations of menopause. These responses suggest that the burden of physical symptoms was their primary concern and that they tended to view menopause as a distressing experience that disrupted daily life. This type was therefore labeled “symptom distress recognition.”

#### Type II: Health management interest

Women in Type II strongly endorsed the following statements: “I became more interested in my health while experiencing menopause” (Z=1.89), “I feel like my self-confidence has decreased since menopause” (Z=1.82), “I have heard health information about menopause in Korea” (Z=1.75), “There are times when accessing healthcare services feels difficult” (Z=1.05), and “I believe that consultations with medical staff are helpful” (Z=1.00).

By contrast, these women disagreed with statements about physical discomfort, including sleep disturbances, fatigue, and vasomotor symptoms. Their responses indicate increased awareness of health management and a strong interest in obtaining health information and professional support. Although they recognized the importance of using health care, they also perceived barriers to access. This type was therefore labeled “health management interest.”

#### Type III: Menopausal acceptance and self-management

Women in Type III expressed strong agreement with “Health education on menopause is necessary” (Z=1.74), “I think menopause is a natural process for women” (Z=1.58), “My joints or muscles ache after menopause” (Z=1.48), “My emotional fluctuations have increased since menopause” (Z=1.38), and “I want to participate in community-based menopause programs” (Z=1.22).

The statements with which they disagreed included “It is difficult to understand menopause due to cultural differences” (Z=–1.15), “More social attention toward menopause is needed” (Z=–1.44), “The experience of menopause serves as an opportunity to reflect on one’s health” (Z=–1.46), “Effective management of menopause can contribute to healthy aging and improve quality of life in later years” (Z=–1.48), and “It is difficult to talk to my family about menopausal symptoms” (Z=–1.95).

These responses suggest that women in this group viewed menopause as a natural life transition and valued menopause-related education and community participation. They reported relatively little difficulty adapting culturally or communicating with family members and were open to acquiring health knowledge. They tended to see menopause as a normal life experience manageable through individual understanding and adaptation, rather than as a major health concern requiring broader social attention. This type was therefore labeled “menopausal acceptance and self-management.”

#### Type IV: Emotional change and social support awareness

Women in Type IV strongly agreed that menopause is a natural process (Z=1.66), that emotional fluctuations had increased since menopause (Z=1.55), that feelings of loneliness or depression occur after menopause (Z=1.33), that menopause needs greater social attention (Z=1.16), and that spousal understanding is important (Z=1.16).

They expressed low agreement with statements emphasizing medical consultation and health care utilization. These responses indicate that the women primarily viewed menopause as an emotional and relational experience rather than a medical condition. They considered social and family support essential to adaptation. This type was therefore labeled “emotional change and social support awareness.”

### Consensus items across types

Despite differences among the four types, the groups agreed on several statements. Across all types, participants positively endorsed “My joints or muscles ache after menopause” (Z=0.90) and “I sometimes feel depressed or lonely after menopause” (Z=0.78) (Table 5).

These responses suggest that multicultural women commonly experience physical discomfort, particularly musculoskeletal pain, and emotional difficulties such as loneliness and depression during menopause. They indicate that certain physical and psychological challenges are universally experienced during the menopausal transition, although individuals differ in their perceptions and coping strategies.

## Discussion

Using Q methodology, this study explored and classified multicultural women’s subjective perceptions of menopausal experiences and health care in Korea. Four distinct types emerged: symptom distress recognition, health management interest, menopausal acceptance and self-management, and emotional change and social support awareness. These findings demonstrate that, for multicultural women, menopause extends beyond a biological event to a multidimensional experience shaped by cultural background, migration experiences, health literacy, social support, and access to health care. They support previous studies that describe the interplay of biological, psychological, social, and cultural influences on immigrant women’s menopausal experiences [9,10].

Women in the symptom distress recognition group were highly aware of physical symptoms, including sleep disturbances, fatigue, and musculoskeletal pain. They regarded these symptoms as burdensome and disruptive to daily life. This finding accords with previous reports that sleep problems, fatigue, and pain are among the most frequent menopausal complaints and are associated with poorer quality of life [1-3]. Despite their symptom awareness, these women showed relatively little interest in health education or active symptom management. This suggests that symptom awareness does not necessarily prompt health-promoting behaviors. Previous studies have likewise reported that limited health literacy and inadequate access to culturally appropriate health information may impede immigrant women’s ability to manage their own health effectively [9,12]. Nursing interventions for this group should therefore address symptom management and improve access to understandable, culturally appropriate health information.

Women in the health management interest group paid greater attention to their health and viewed professional consultation positively. They valued obtaining health information and seeking help from health care professionals. However, they also reported difficulty accessing health care services. This finding reflects barriers commonly encountered by immigrant women: language difficulties, unfamiliarity with health care systems, and limited communication with providers [6-8]. Previous research has documented similar difficulties in navigating services and obtaining appropriate health information [18,19]. The combination of high health awareness and limited access in this group suggests that improving access to culturally responsive health care is as important as providing health education. Multilingual educational materials, interpretation services, and culturally sensitive counseling may help overcome these barriers.

Women in the menopausal acceptance and self-management group viewed menopause as a natural life transition and actively engaged in health-promoting behaviors. They strongly supported menopause-related education and were willing to participate in community-based health programs. These findings align with previous reports associating positive attitudes toward menopause with greater engagement in self-care and health-promoting behaviors [20,21]. The women regarded menopause as an expected, manageable stage of life rather than a crisis. Their proactive approach suggests that they may benefit particularly from community-based health promotion programs designed to strengthen self-efficacy and maintain healthy lifestyles in midlife [20,21].

Women in the emotional change and social support awareness group placed emphasis on emotional experiences and interpersonal relationships. They reported emotional fluctuations, loneliness, and depressive feelings and attached considerable importance to support from spouses and family members. Previous studies have consistently shown that social support and interpersonal relationships strongly influence emotional well-being during menopause [7,11]. Migration and cultural adaptation may restrict multicultural women’s social networks, so family support may be especially important for healthy adjustment. These findings suggest that interventions for this group should address emotional support, family-centered education, and stronger community connections alongside symptom management.

Musculoskeletal pain and feelings of loneliness or depression were consistently reported across all four types. Agreement with these consensus statements indicates that multicultural women share certain physical and emotional challenges despite differences in subjective perception. Previous studies have likewise identified musculoskeletal discomfort and emotional distress among the most prevalent menopausal symptoms across diverse cultural contexts [3,7,20]. Although cultural beliefs and personal experiences may shape symptom interpretation and management, these shared symptoms suggest common health needs that menopause-related health care services should address. Participants placed greater emphasis on musculoskeletal symptoms, such as joint and muscle pain, than on classic vasomotor symptoms, such as hot flashes and night sweats. Cross-cultural comparisons have also found substantial ethnic variation in the prevalence and salience of vasomotor and somatic symptoms. Asian women generally report fewer vasomotor symptoms and more musculoskeletal or somatic complaints than women from other ethnic backgrounds, a pattern attributed to ethnic differences in estrogen metabolism, dietary patterns, and culturally influenced symptom reporting [2,22,23]. Because the participants also had to adapt to Korean dietary and lifestyle patterns after migration, both ethnic and acculturation-related differences in diet and lifestyle may have contributed to their symptom profile. For example, shifts away from diets traditionally rich in soy and phytoestrogens, changes in physical activity, and reduced sun exposure and vitamin D synthesis after resettlement could plausibly affect circulating estrogen levels and musculoskeletal health, thereby altering the relative prominence of vasomotor and musculoskeletal symptoms [24,25]. Future research should examine this pathway directly.

The findings also suggest that universal biological changes and culturally specific interpretations interact to shape multicultural women’s menopausal experiences. Even when women experience similar symptoms, the meanings they attach to them may vary with cultural values, migration experiences, health beliefs, and social environments [10,21,26,27]. Health care providers should therefore recognize this variation and avoid assuming that one intervention strategy will work for all women. Interventions should instead reflect each perception type’s characteristics and needs.

The study’s implications for women’s health nursing practice include several areas for action. First, nursing interventions that are sensitive to cultural differences should be developed to address multicultural women’s diverse perceptions and health needs [28]. Second, multilingual resources, interpretation services, and culturally appropriate educational materials should be provided to improve access to health care. Third, family-centered and community-based support programs should be expanded to address the emotional and social challenges of menopause. Integrating these strategies can help health care professionals better support multicultural women’s health and quality of life during the menopausal transition.

The findings indicate that multicultural women’s menopausal experiences and perceptions of menopause-related health care involve physical, psychological, social, and cultural dimensions. Identifying four distinct perception types provides evidence to inform tailored nursing interventions, culturally responsive health education, and policies promoting health equity for multicultural women experiencing menopause.

The study has several limitations. Its relatively small sample was drawn from a limited number of geographic regions, and Q methodology identifies patterns of subjectivity rather than population-level prevalence; the findings should therefore not be generalized to all multicultural women. Future research should recruit participants from more diverse cultural and regional backgrounds and evaluate type-specific intervention programs informed by these findings. In conclusion, multicultural women’s menopausal experiences and health care perceptions form a complex, multidimensional phenomenon shaped by biological, psychological, social, and cultural factors. Tailored, culturally responsive nursing interventions and health policies are needed to improve this population’s health outcomes and quality of life during the menopausal transition.

## Figures and Tables

**Figure 1.**
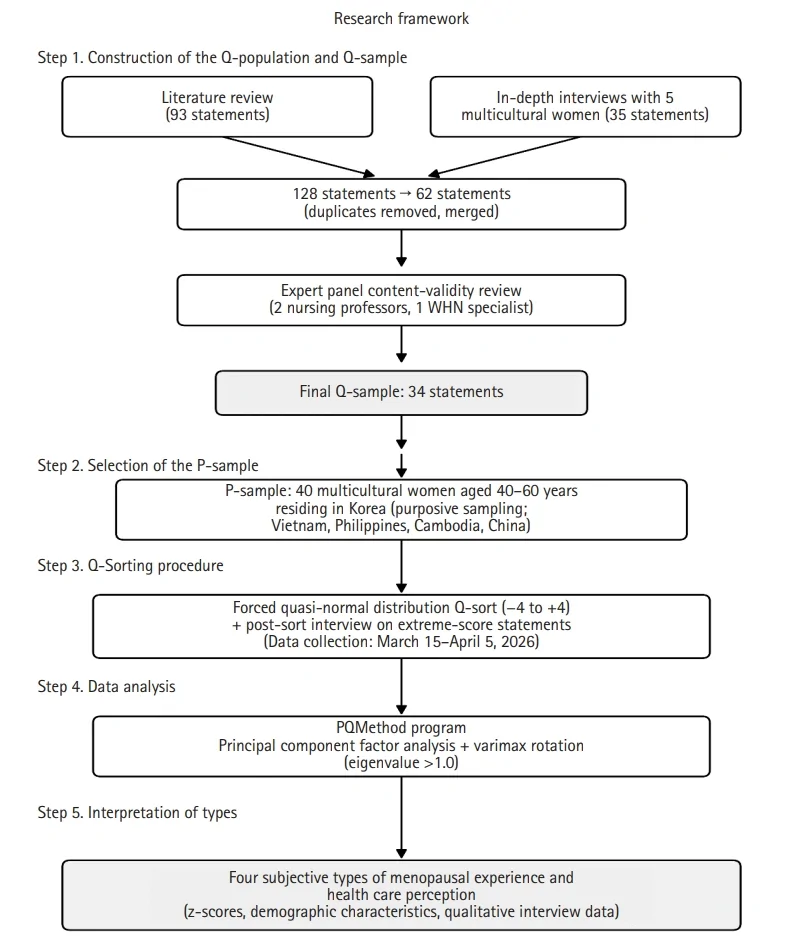
Study framework. P-sample: Participant sample; Q-sample: set of Q-statements used for Q-sorting; WHN: women’s health nursing. PQMethod ver. 2.35 (Peter Schmolck, Neubiberg, Germany).

**Table 1.** Participant characteristics and factor weights (N=40)

Type	ID	Factor weight	Age (yr)	Nationality	Years in Korea	Years married	Employed	Menopausal symptoms	Health info.	Care use	Family support
Type I (n=13)	2	0.99	45–49	Filipino	10–15	10–15	Yes	Not sure	No	No	Not at all
	3	2.14	50–54	Filipino	10–15	≥15	Yes	Not sure	No	Yes	Very high
	4	1.13	40–44	Vietnamese	5–10	5–10	No	No	No	Yes	Very high
	8	0.34	40–44	Vietnamese	5–10	5–10	No	No	No	Yes	Very high
	15	0.95	45–49	Filipino	10–15	5–10	No	No	No	Yes	Not at all
	16	0.66	40–44	Vietnamese	10–15	5–10	No	Not sure	Yes	No	Not at all
	17	1.21	45–49	Filipino	10–15	5–10	No	Not sure	Yes	Yes	Very high
	24	1.57	40–44	Vietnamese	5–10	5–10	No	No	No	Yes	Very high
	25	1.01	40–44	Vietnamese	5–10	5–10	No	No	No	Yes	Very high
	28	0.76	40–44	Chinese	5–10	5–10	Yes	Yes	No	Yes	Moderate
	29	0.61	45–49	Filipino	10–15	≥15	Yes	Yes	No	Yes	Moderate
	32	1.25	50–54	Cambodian	10–15	5–10	No	No	No	Yes	Very high
	40	1.53	40–44	Vietnamese	5–10	10–15	Yes	Yes	No	Yes	Moderate
Type II (n=11)	5	0.23	40–44	Vietnamese	5–10	≥15	No	No	No	Yes	Very high
	10	0.47	45–49	Filipino	10–15	10–15	No	No	Yes	Yes	Moderate
	12	0.51	50–54	Filipino	5–10	10–15	No	Not sure	Yes	Yes	Not at all
	13	0.62	50–54	Filipino	≥15	10–15	No	No	Yes	No	Not at all
	19	0.50	40–44	Vietnamese	5–10	≥15	No	No	No	Yes	Moderate
	20	0.82	45–49	Filipino	10–15	5–10	Yes	Yes	No	Yes	Very high
	23	0.54	50–54	Filipino	5–10	10–15	No	Not sure	Yes	Yes	Not at all
	26	0.96	50–54	Filipino	≥15	10–15	No	No	Yes	No	Not at all
	35	0.57	45–49	Filipino	10–15	≥15	No	No	No	No	Not at all
	37	0.16	40–44	Vietnamese	5–10	5–10	No	No	No	Yes	Moderate
	38	0.94	40–44	Vietnamese	<5	5–10	No	No	Yes	Yes	Moderate
Type III (n=9)	6	0.90	40–44	Vietnamese	<5	5–10	No	No	Yes	Yes	Very high
	9	0.56	45–49	Filipino	10–15	≥15	Yes	Yes	No	Yes	Moderate
	11	0.74	40–44	Vietnamese	5–10	10–15	No	No	No	Yes	Moderate
	18	0.84	45–49	Vietnamese	10–15	≥15	No	No	Yes	Yes	Not at all
	21	0.91	40–44	Vietnamese	10–15	5–10	No	No	Yes	No	Not at all
	22	0.18	50–54	Cambodian	10–15	5–10	No	No	No	Yes	Very high
	27	1.19	50–54	Filipino	≥15	≥15	Yes	Yes	Yes	Yes	Not at all
	31	1.25	40–44	Vietnamese	5–10	5–10	No	No	No	Yes	Very high
	33	0.90	40–44	Vietnamese	10–15	5–10	No	No	No	Yes	Very high
Type IV (n=7)	1	0.51	≥55	Cambodian	≥15	10–15	No	No	No	No	Not at all
	7	0.56	40–44	Vietnamese	5–10	5–10	Yes	Yes	Yes	Yes	Not at all
	14	1.20	≥55	Cambodian	10–15	5–10	No	No	No	No	Not at all
	30	0.89	50–54	Vietnamese	10–15	≥15	No	No	Yes	Yes	Not at all
	34	0.23	40–44	Filipino	5–10	5–10	No	Not sure	No	Yes	Not at all
	36	0.59	50–54	Cambodian	≥15	5–10	No	No	No	No	Not at all
	39	0.54	50–54	Chinese	10–15	≥15	No	No	Yes	Yes	Not at all

ID: Participant identification number; Health info.: receipt of menopausal health information; Care use: use of hospitals or public health centers for health concerns. Type I: Symptom distress recognition; Type II: health management interest; Type III: menopausal acceptance and self-management; Type IV: emotional change and social support awareness.

**Table 2.** Eigenvalues, variance, and cumulative variance (N=40)

Variable	Type I	Type II	Type III	Type IV
Eigenvalue	7.54	4.40	3.45	2.61
Variance (%)	18.85	11.00	8.63	6.52
Cumulative variance (%)	18.85	29.85	38.48	44.99

Type I: Symptom distress recognition; Type II: health management interest; Type III: menopausal acceptance and self-management; Type IV: emotional change and social support awareness.

**Table 3.** Correlations among perception types (N=40)

Type	r (*p*)			
	Type I	Type II	Type III	Type IV
Type I	1.000			
Type II	.12 (.49)	1.000		
Type III	.23 (.19)	.02 (.91)	1.000	
Type IV	–.07 (.71)	–.05 (.78)	–.10 (.58)	1.000

Type I: Symptom distress recognition; Type II: health management interest; Type III: menopausal acceptance and self-management; Type IV: emotional change and social support awareness.

**Table 4.** Representative Q-statements and Z-scores by perception type (N=40)

Type	n	Statement	Z-score
Type I: Symptom distress recognition	13	3. It is difficult to sleep well after menopause	2.13
		4. Menopausal symptoms sometimes make daily life difficult	1.94
		5. My joints or muscles ache after menopause	1.60
		2. I get tired easily after menopause	1.36
		6. I think menopause is a natural process for women	1.33
		24. Health education on menopause is necessary	–1.21
		32. Menopause is a process that should be accepted naturally	–1.26
		31. More social attention toward menopause is needed	–1.37
		33. Effective management of menopause can contribute to healthy aging and improve quality of life in later years	–1.55
		34. The experience of menopause serves as an opportunity to reflect on one’s health	–1.61
Type II: Health management interest	11	11. I became more interested in my health while experiencing menopause	1.89
		10. I feel like my self-confidence has decreased since menopause	1.82
		12. I have heard health information about menopause in Korea	1.75
		14. There are times when accessing healthcare services feels difficult	1.05
		26. I believe that consultations with medical staff are helpful	1.00
		30. Talking about menopause with people around me is helpful	–1.12
		3. It is difficult to sleep well after menopause	–1.52
		2. I get tired easily after menopause	–1.82
		1. My face suddenly flushes or I sweat after menopause	–2.18
		23. I think getting enough rest is helpful	–2.28
Type III: Menopausal acceptance and self-management	9	24. Health education on menopause is necessary	1.74
		6. I think menopause is a natural process for women	1.58
		5. My joints or muscles ache after menopause	1.48
		7. My emotional fluctuations have increased since menopause	1.38
		25. I want to participate in community-based menopause programs	1.22
		17. It is difficult to understand menopause due to cultural differences	–1.15
		31. Greater social attention toward menopause is needed	–1.44
		34. The experience of menopause serves as an opportunity to reflect on one’s health	–1.46
		33. Effective management of menopause can contribute to healthy aging and improve quality of life in later years	–1.48
		15. It is difficult to talk to my family about menopausal symptoms	–1.95
Type IV: Emotional change and social support awareness	7	6. I think menopause is a natural process for women	1.66
		7. My emotional fluctuations have increased since menopause	1.55
		8. I sometimes feel depressed or lonely after menopause	1.33
		31. Greater social attention toward menopause is needed	1.16
		29. Spousal understanding is important	1.16
		19. Dietary adjustments are made for menopausal health	–1.17
		1. My face suddenly flushes or I sweat after menopause	–1.32
		20. I think it is good to go to the hospital if there are menopausal symptoms	–1.80
		27. It is difficult to obtain information about menopause	–1.89
		26. I believe that consultations with medical staff are helpful	–2.17

**Table 5.** Consensus items and mean Z-scores (N=40)

Q-statement	Z-score
Q5. My joints or muscles ache after menopause	0.90
Q8. I sometimes feel depressed or lonely after menopause	0.78
